# Brazilian epidemiology facing new challenges: the Fifth Strategic Plan and the renewal of an academic, social, and political project

**DOI:** 10.1590/1980-549720250007.supl.1

**Published:** 2026-07-24

**Authors:** Tânia Maria de Araújo, Ana Paula Muraro, Alexandra Crispim Boing, Maria Rita Donalisio

**Affiliations:** I Universidade Estadual de Feira de Santana, Center for Epidemiology – Feira de Santana (BA), Brazil.; II Universidade Federal de Mato Grosso, Collective Health Institute – Cuiabá (MT), Brazil.; III Universidade Federal de Santa Catarina, Florianópolis (SC), Brazil.; IV Universidade Estadual de Campinas, School of Medical Sciences – Campinas (SP), Brazil.

The field of epidemiology in Brazil emerges from a long trajectory of organization and solid construction, sustained by efforts to forge a critical and proactive field. In this context, the 5th Strategic Plan for the Development of Epidemiology in Brazil represents a strategic milestone, guiding the advancement of the field and strengthening its historical commitments.

The creation of the Brazilian Association of Collective Health (Abrasco) in 1979 constituted a foundational step in the reconfiguration of struggles for democracy and health rights in the country, articulating broad mobilizations within the health sector with political and social movements of resistance and struggle against the military dictatorship^1^. Brazilian epidemiologists participated actively in the process of building and consolidating both Abrasco and the field of collective health in the country^2^. In 1984, the 1st National Meeting on Teaching and Research in Epidemiology took place, during which participants instituted Abrasco’s Epidemiology Commission.

The development of strategic plans for epidemiology in Brazil unfolded across various moments of collective reflection and planning. In May 1989, in Itaparica (BA), the seminar “Strategies for the development of epidemiology in Brazil” was held; this event marked the beginning of a structured process of discussion regarding the pathways and priorities for strengthening epidemiology in the country. This strategic plan^3^ focused on the relationship between living conditions and health status, identifying the primary health problems affecting the Brazilian population and outlining a set of measures to address them. The implementation of this plan proved to be a promising road toward achieving the necessary advancements. Thus, in April 1994, in Olinda (Pernambuco), the seminar “Directions of Brazilian Epidemiology: National Meeting on Assessment and Perspectives” was held. This event analyzed the progress achieved and discussed new perspectives for the field, giving rise to the 2nd Strategic Plan^4^. The 3rd Strategic Plan^5^ was drafted in Brasília in August 2000 and strengthened the collaboration between academic institutions and government agencies, seeking to consolidate strategies for bolstering epidemiology within the Brazilian health system. The 4th Strategic Plan^6^ was discussed during the seminar “Definition of Guidelines and Proposals,” held in Rio de Janeiro in 2005. This event continued the strategic planning process for the field, contributing to the updating of guidelines and steering new initiatives aimed at the development of epidemiology in Brazil.

In addition to fostering significant consensus by defining priority problems and strategic actions, these plans have driven substantial progress - most notably regarding initiatives to consolidate research and graduate-level studies in collective health in the country. The resulting legacy is both broad and diverse. Prominent among these initiatives are the hosting of the 1st Brazilian Congress of Epidemiology (Campinas, SP), a proposal stemming from the 1st Strategic Plan of 1990, and the establishment of the Revista Brasileira de Epidemiologia (Brazilian Journal of Epidemiology), which was proposed in the 2nd Strategic Plan and launched in 1998. Other significant initiatives — such as the creation of the National Center for Epidemiology (CENEPI) in 1990; the National Exhibition of Successful Experiences in Epidemiology, Disease Prevention, and Control (ExpoEpi) in 2001; and the Secretariat of Health Surveillance in 2003 — also aligned closely with the proposals emerging from these Strategic Plans.

This 5th Strategic Plan seeks to revive the tradition of strategic organization, innovation, and collaborative capacity that has historically characterized the development of epidemiology in Brazil. Its formulation takes place within a challenging context, which is marked by the persistence and deepening of social inequalities, the spread of scientific denialism, the emergence and re-emergence of health threats, the climate crisis, and the persistent underfunding of public policies. These processes impose new and complex challenges upon the field of epidemiology, necessitating a critical review of its agendas, methods, and intervention strategies. Furthermore, they demand a strengthening of its commitment to producing socially relevant knowledge — knowledge specifically oriented toward the analysis, monitoring, and mitigation of social inequalities in health. The development of the 5th Strategic Plan unfolded across multiple stages, including online surveys, debates, and in-person workshops. Workshop I (Brasília, 2023) identified priority challenges, while Workshop II (Rio de Janeiro, 2024) defined strategies for addressing them. Characterized by a participatory and democratic nature, the drafting process benefited from the expert contributions of epidemiologists from all regions of the country, guided by principles of plurality, diversity, and the promotion of gender and race/ethnicity equity.

This supplement of the Revista Brasileira de Epidemiologia aims to disseminate the 5th Strategic Plan for the Development of Epidemiology in Brazil (2025–2029), covering the three major thematic areas in the field of epidemiology: education; research; and health policies, programs, and services. In addition to the full text of the 5th Strategic Plan, three articles delve into the key points of debate within each of these three major areas.

The article “Epidemiology in health policies, programs, and services in Brazil: trajectories and perspectives of the 5th Strategic Plan for the development of epidemiology in Brazil (2025–2029)” by Abrasco^8^ highlights the limitations and potential of national health information systems. Included are the challenges related to the production of high-quality data and information, the dilemmas surrounding socially and politically engaged epidemiology education, the need to broaden the scope of health surveillance, the relevance of equity-oriented evaluation processes, and intersectoral and territorial coordination as a strategy for strengthening the field. The article “Epidemiology research in Brazil: challenges, paths, and commitments for the future^9^” analyzes the advancements in epidemiological research over time and identifies its primary challenges such as chronic underfunding, inequalities in resource distribution, academic productivism, the need to bridge the gap between science and society, and the strengthening of the theoretical-methodological foundation of studies. The text also discusses topics such as open science, the democratization of data access, and the emphasis on assessing social inequalities in health. Epidemiology education in Brazil is the subject of the article “Critical mapping of epidemiology education within collective health post-graduate programs in Brazil: challenges”^10^. Based on documentary survey data from graduate programs in Collective Health and a survey of faculty members and students in these programs, the curricular proposals, pedagogical practices, and institutional conditions shaping training in Epidemiology are critically analyzed. The objective is to provide a diagnostic assessment of teaching in this field, identifying its characteristics, strengths, and weaknesses. These articles present reflections stemming from the process of formulating the plan, thereby facilitating a deeper exploration of aspects crucial to addressing the challenges in each of the thematic areas.

This supplement also includes articles on themes that were widely discussed during the development of the 5th Strategic Plan and at the 12th Brazilian Congress of Epidemiology (2024). These articles address epidemiology’s place within the field of collective health — specifically its interactions, dialogues, and tensions with the other foundational fields of collective health (Epidemiology in dialogue: belonging to the field of collective health, practices, and theoretical tensions)^11^ — as well as epidemiology’s specific contributions to health surveillance (Epidemiology and health surveillance: synergies and challenges in a changing world)^12^. The analytical integration of race/color as an essential contribution to the advancement of epidemiology is presented in the article “Unveiling inequities: the role of epidemiology in combating racism, a contribution that broadens our understanding of health inequalities and the social determinants associated with structural racism.

These articles underscore the relevance of a critical, ethical, and socially committed epidemiology, one that is actively engaged in the defense of human rights and contributes to strengthening an agenda for action dedicated to quality, innovation, equity, and social transformation.

The launch of the 5th Strategic Plan took place at the 12th Brazilian Congress of Epidemiology (2024), thereby resuming the ongoing trajectory of planning and continuous review of our practices and goal-setting processes. In 2025, the Epidemiology Commission organized a dissemination strategy featuring launch events and activities across all five regions of the country. This supplement constitutes part of those dissemination efforts. The challenge that now lies ahead is the Plan’s implementation.

The 5th Strategic Plan establishes guidelines for the coming years. Its implementation is contingent upon collective efforts coordinated among undergraduate and graduate programs in collective health, researchers, health professionals, policymakers, and administrators, stakeholders whose respective responsibilities and contributions complement one another in addressing and confronting the challenges identified herein. The Plan aims to strengthen the training of professionals who are critical, reflective, and socially committed, technically well-prepared, and capable of navigating new healthcare landscapes. These include the impacts of the climate crisis, shifts in disease profiles, challenges regarding mental and occupational health, the integration of technologies such as artificial intelligence, and scientific communication and dissemination. Furthermore, the Plan seeks to bolster information systems and innovative health surveillance strategies; to stimulate research into social determinants, emerging health threats, and inequalities; and to guide practices that are attuned to the real health problems of the population and respectful of traditional knowledge.

In this regard, the commitment of Brazilian epidemiology to democracy and to the defense of health as a fundamental right is reaffirmed in this 5th Strategic Plan. Integrated and proactive collaboration between various stakeholders is essential for improving health indicators, consolidating epidemiology as a strategic tool for addressing inequalities, strengthening the Unified Health System (SUS), and fostering closer alignment between knowledge production and societal needs.
