# Supplementary material for: Brazilian epidemiology facing new challenges: the Fifth Strategic Plan and the renewal of an academic, social, and political project
Source: Rev Bras Epidemiol. 2026 Jul 24;28(Suppl 1):e250007supl1. doi: 10.1590/1980-549720250007.supl.1 (PMC13405551; doi:10.1590/1980-549720250007.supl.1)
Supplement: Translation

# Translation: Epidemiologia brasileira diante de novos desafios: o V Plano Diretor e a renovação de um projeto acadêmico, social e político

**DOI:** 10.1590/1980-549720250007.supl.1.2

**Authors:** Tânia Maria de Araújo, Ana Paula Muraro, Alexandra Crispim Boing, Maria Rita Donalisio

**Affiliations:** I Universidade Estadual de Feira de Santana, Núcleo de Epidemiologia – Feira de Santana (BA), Brasil.; II Universidade Federal de Mato Grosso, Instituto de Saúde Coletiva – Cuiabá (MT), Brasil.; III Universidade Federal de Santa Catarina, Florianópolis (SC), Brasil.; IV Universidade Estadual de Campinas, Faculdade de Ciências Médicas – Campinas (SP), Brasil.

O campo da epidemiologia no Brasil emerge de uma longa trajetória de organização e construção sólida, sustentada por esforços para formar um campo crítico e proativo. Nesse contexto, o V Plano Diretor para o Desenvolvimento da Epidemiologia no Brasil (2025–2029) representa um marco estratégico, orientando o avanço da área e fortalecendo seus compromissos históricos.

A criação da Associação Brasileira de Saúde Coletiva (Abrasco), em 1979, foi um passo estruturante de reconfiguração das lutas por democracia e direitos à saúde no país, articulando amplas mobilizações no âmbito da saúde, com movimentos políticos e sociais de resistência e de luta contra a ditadura militar^1^. Os(as) epidemiologistas brasileiros(as) participaram ativamente do processo de construção e consolidação da Abrasco e do campo da saúde coletiva no país^2^. Em 1984, ocorreu a I Reunião Nacional sobre Ensino e Pesquisa em Epidemiologia, ocasião em que foi criada a Comissão de Epidemiologia da Abrasco.

A construção dos planos diretores para o desenvolvimento da epidemiologia no Brasil ocorreu ao longo de diferentes momentos de reflexão e planejamento coletivo. Em maio de 1989, em Itaparica (BA), foi realizado o Seminário “Estratégias para o desenvolvimento da epidemiologia no Brasil”, que marcou o início de um processo estruturado de discussão sobre os caminhos e as prioridades para fortalecer a epidemiologia no país. Esse Plano Diretor^3^ focalizou a relação entre condições de vida e a situação de saúde, identificando os principais problemas de saúde da população brasileira e traçando um conjunto de medidas para o seu enfrentamento. A implementação desse Plano revelou-se um caminho promissor para os avanços necessários. Assim, em abril de 1994, em Olinda (PE), foi realizado o Seminário “Rumos da epidemiologia brasileira: reunião nacional de avaliação e perspectivas”. O evento analisou os avanços alcançados e discutiu novas perspectivas para o campo, originando o II Plano Diretor^4^. O III Plano Diretor^5^ foi elaborado em Brasília, em agosto de 2000, e reforçou a articulação entre instituições acadêmicas *e* órgãos governamentais, buscando consolidar estratégias para o fortalecimento da epidemiologia no sistema de saúde brasileiro. O IV Plano^6^ foi discutido no seminário “Definição de diretrizes e propostas”, realizado na cidade do Rio de Janeiro, em 2005. Esse momento deu continuidade ao processo de planejamento estratégico da área, contribuindo para atualizar diretrizes e orientar novas ações voltadas ao desenvolvimento da epidemiologia no Brasil.

Esses Planos, além de criar consensos relevantes ao definir problemas prioritários e ações estratégicas, impulsionaram avanços significativos, com destaque para ações de consolidação da pesquisa e da Pós-Graduação em Saúde Coletiva no país. O legado deixado é amplo e diverso. Entre as iniciativas, destacam-se a realização do I Congresso Brasileiro de Epidemiologia (Campinas – SP), uma proposição do I Plano, de 1990, e a criação da Revista Brasileira de Epidemiologia, proposta no II Plano e concretizada em 1998. Iniciativas relevantes, como a criação do Centro Nacional de Epidemiologia (CENEPI), em 1990; da Mostra Nacional de Experiências Bem-sucedidas em Epidemiologia, Prevenção e Controle de Doenças (ExpoEpi), em 2001; e da Secretaria de Vigilância em Saúde, em 2003, também dialogaram com as proposições emergentes nos Planos Diretores.

Este V Plano busca retomar a tradição de organização estratégica, inovação e capacidade de articulação que historicamente marcaram o desenvolvimento da epidemiologia no Brasil. Sua formulação ocorre em um contexto desafiador, marcado pela persistência e pelo aprofundamento das desigualdades sociais; pela disseminação do negacionismo científico; pela emergência e reemergência de agravos à saúde; pela crise climática; e pelo persistente desfinanciamento de políticas públicas. Tais processos impõem novos e complexos desafios para a epidemiologia, exigindo a revisão crítica de suas agendas, métodos e formas de intervenção, bem como demanda o fortalecimento de seu compromisso com a produção de conhecimento socialmente referenciado e orientado à análise, ao monitoramento e ao enfrentamento das desigualdades sociais em saúde. A elaboração do V Plano Diretor para o Desenvolvimento da Epidemiologia no Brasil (2025–2029) ocorreu em múltiplas etapas, incluindo inquéritos *online*, debates e oficinas presenciais. A Oficina I (Brasília, 2023) identificou desafios prioritários e a Oficina II (Rio de Janeiro, 2024) definiu estratégias de enfrentamento. Marcado por um caráter participativo e democrático, o processo de elaboração contou com a contribuição qualificada de epidemiologistas de todas as regiões do país, orientando-se por princípios de pluralidade, diversidade e promoção da equidade de gênero e raça/etnia.

Este Suplemento da Revista Brasileira de Epidemiologia tem a finalidade de divulgar o V Plano Diretor para o Desenvolvimento da Epidemiologia no Brasil 2025–2029^7^, contemplando as três grandes áreas temáticas do campo da Epidemiologia: Formação; Pesquisa; e Políticas, Programas e Serviços de Saúde. Além do texto completo do V Plano Diretor para o Desenvolvimento da Epidemiologia no Brasil (2025–2029), três artigos aprofundam os principais pontos de debate em cada uma dessas três grandes áreas.

O artigo Epidemiologia em políticas, programas e serviços de saúde no Brasil: trajetórias e perspectivas do V Plano Diretor para o Desenvolvimento da Epidemiologia no Brasil (2025–2029) da Abrasco^8^ destaca as limitações e potencialidades dos sistemas nacionais de informação em saúde; os desafios relacionados à produção de dados e informações qualificadas; os dilemas da formação em epidemiologia comprometida social e politicamente; e a necessidade de ampliar os sentidos da vigilância em saúde, a relevância de processos avaliativos orientados pela equidade e a articulação intersetorial e territorial como estratégia de fortalecimento do campo. O artigo Pesquisa em epidemiologia no Brasil: desafios, caminhos e compromissos para o futuro^9^ analisa os avanços da pesquisa epidemiológica ao longo do tempo e aponta os seus principais desafios, como o subfinanciamento crônico, as desigualdades na distribuição de recursos, o produtivismo acadêmico, a necessidade de aproximar a ciência da sociedade e o fortalecimento da base teórico-metodológica dos estudos. O texto também discute temas como a ciência aberta, a democratização do acesso a dados e o foco na avaliação das desigualdades sociais em saúde. A formação em Epidemiologia no Brasil é objeto do artigo Mapeamento crítico da formação em epidemiologia nos programas de pós-graduação em Saúde Coletiva no Brasil: desafios^10^. A partir de dados de levantamento documental dos programas de pós-graduação de saúde coletiva e de um inquérito com docentes e discentes desses programas, são analisadas criticamente as propostas curriculares, as práticas pedagógicas e as condições institucionais que conformam a formação em Epidemiologia, com o objetivo de traçar um diagnóstico do ensino da área, identificando suas características, forças e fragilidades. Esses artigos desenvolvem reflexões oriundas no processo de elaboração do Plano, permitindo o aprofundamento de aspectos cruciais ao enfrentamento dos desafios em cada uma das áreas temáticas.

Neste Suplemento, também estão incluídos artigos sobre temas amplamente discutidos na elaboração do V Plano Diretor para o Desenvolvimento da Epidemiologia no Brasil (2025–2029) e no 12° Congresso Brasileiro de Epidemiologia (2024). Esses artigos abordam o pertencimento da epidemiologia ao campo da saúde coletiva e sua interlocução, seus diálogos e suas tensões com os demais campos formativos da saúde coletiva (Epidemiologia em diálogos: pertencimento no campo da saúde coletiva, práticas e tensionamentos teóricos)^11^; e as contribuições específicas da Epidemiologia para a Vigilância em Saúde (Epidemiologia e vigilância em saúde: sinergias e desafios em um mundo em transformação)^12^. A incorporação analítica da raça/cor como aporte fundamental ao desenvolvimento da epidemiologia é apresentada no artigo “Desvelando iniquidades: o papel da epidemiologia no enfrentamento do racismo”, uma contribuição que amplia a compreensão das desigualdades em saúde e das determinações sociais associadas ao racismo estrutural.

Esses artigos evidenciam a relevância de uma epidemiologia crítica, ética, socialmente comprometida e engajada na defesa dos direitos humanos, e contribuem para fortalecer uma agenda de atuação comprometida com a qualidade, a inovação, a equidade e a transformação social.

O lançamento do V Plano Diretor para o Desenvolvimento da Epidemiologia no Brasil (2025–2029) ocorreu no 12° Congresso Brasileiro de Epidemiologia (2024), retomando a trajetória de planejamento e revisão contínua das nossas práticas e planejamento de metas. Em 2025, a Comissão de Epidemiologia estruturou a sua divulgação com lançamentos e atividades nas cinco regiões do país. Este Suplemento é parte desses esforços de divulgação. O desafio que se apresenta agora é a sua implementação.

O V Plano Diretor para o Desenvolvimento da Epidemiologia no Brasil (2025–2029) estabelece diretrizes para os próximos anos. Sua implementação está condicionada aos esforços coletivos articulados entre a graduação e pós-graduação em saúde coletiva, pesquisadores(as), profissionais de saúde, formuladores(as) de políticas e gestores(as), cujas responsabilidades e contribuições se complementam na abordagem e no enfrentamento dos desafios identificados. O Plano visa fortalecer a formação de profissionais críticos, reflexivos e socialmente comprometidos, tecnicamente bem-preparados, capazes de lidar com os novos cenários da saúde, incluindo os impactos da crise climática, as transformações nos perfis de adoecimento, os desafios à saúde mental e do(a) trabalhador(a), a incorporação de tecnologias como a inteligência artificial e a comunicação e divulgação científica. Busca-se, ainda, fortalecer sistemas de informação e estratégias inovadoras de vigilância em saúde, estimular pesquisas sobre determinantes sociais, agravos emergentes e desigualdades, bem como orientar práticas sintonizadas com os problemas reais da população e respeitosas aos saberes populares.

Nesse sentido, o compromisso da epidemiologia brasileira com a democracia e com a defesa da saúde como direito é reafirmado nesse V Plano Diretor para o Desenvolvimento da Epidemiologia no Brasil (2025–2029). A articulação integrada e proativa entre os diferentes atores é fundamental para promover a melhoria dos indicadores de saúde e consolidar a epidemiologia como instrumento estratégico para enfrentar desigualdades, fortalecer o Sistema Único de Saúde e intensificar a aproximação entre a produção de conhecimento e as demandas da sociedade.
